# Tendril extract of *Cucurbita moschata* suppresses NLRP3 inflammasome activation in murine macrophages and human trophoblast cells

**DOI:** 10.7150/ijms.39003

**Published:** 2020-04-06

**Authors:** Ji-Yeon Park, Sung-Gang Jo, Ha-Nul Lee, Joo-Hee Choi, Yeon-Ji Lee, Young-Min Kim, Jeong-Yong Cho, Sung Ki Lee, Jong-Hwan Park

**Affiliations:** 1 Laboratory Animal Medicine, College of Veterinary Medicine and Animal Medical Institute, Chonnam National University, Gwangju, Republic of Korea; 2 Department of Food Science & Technology, Chonnam National University, Gwangju, Republic of Korea; 3 Laboratory Animal Center, Daegu-Gyeongbuk Medical Innovation Foundation, Daegu, Republic of Korea; 4 Department of Obstetrics and Gynecology, College of Medicine, Konyang University, Daejeon, Republic of Korea

**Keywords:** tendril of *Cucurbita moschata* Duch, NLRP3 inflammasome, bone marrow derived macrophage, trophoblast, SW.71

## Abstract

Inflammation is the root cause of many diseases that pose a serious threat to human health. Excessive inflammation can also result in preterm birth or miscarriage in pregnant women. Pumpkin (*Cucurbita moschata* Duchesne, CMD) is a well-known traditional health food and medicinal herb used in many countries to treat diabetes, obesity, osteoporosis, cancer and other diseases. In this study, we investigated the effects of hot water extract derived from the tendrils of *C. moschata* Duchesne (TCMD) on NLRP3 inflammasome activation in murine macrophages and human trophoblast cells. The TCMD treatment of LPS-primed bone marrow-derived macrophages (BMDMs) and human trophoblast cells attenuated NLRP3 inflammasome activation induced by inflammasome activators such as ATP, nigericin, and monosodium urate (MSU). TCMD treatment suppressed IL-1β secretion in a dose-dependent manner, without affecting IL-6 secretion. In addition, TCMD inhibited NLRP3-dependent pyroptosis in BMDMs. TCMD also suppressed the release of mature IL-1β and activation of cleaved-caspase-1 via limited ASC oligomerization. Furthermore, TCMD significantly inhibited IL-1β secretion and pyroptotic cell death in human trophoblast cells. These results suggest that TCMD exhibits anti-inflammatory effects mediated via inhibition of NLRP3 inflammasome activation suggesting therapeutic potential against inflammatory diseases, preterm birth, and miscarriage.

## Introduction

Inflammation is one of the cellular defenses against deleterious stimuli such as tissue damage, infection, and exposure to toxic agents, and is strictly regulated by macrophages responsible for production of inflammatory molecules 1, 2. In this systemic inflammatory response, macrophages produce inflammatory mediators such as NO, IL-6, TNF-a, and IL-1, which act as biological defenses in early infection 3. However, persistent inflammatory response due to the overproduction of these mediators promotes mucosal injury, and as a result, leads to chronic inflammatory diseases such as arteriosclerosis, diabetes, arthritis, and cancer 4. Intrauterine inflammation also causes spontaneous preterm birth and triggers various perinatal complications in premature infants including mortality 5. However, therapeutic control of inflammation in preterm birth remains undeveloped. Control of intrauterine inflammatory response is an effective treatment strategy for prevention of preterm birth.

The NLRP3 inflammasome is the most studied inflammasome. The NLRP3 inflammasome is composed of NLRP3, caspase-1, and ASC, and assembled into an active protein complex in response to both microbial infection and endogenous “danger signal” 6-8. The caspase-1 is activated after the activation of the NLRP3 inflammasome. Activated caspase-1 leads to cleavage of the precursor forms of the pro-IL-1β, which mediate the inflammatory response 9-14. However, excessive inflammation via NLRP3 activation results in serious diseases such as arthritis, asthma, inflammatory bowel disease (IBD), Parkinson's disease, Alzheimer's disease, and sepsis 15-22. Preterm delivery is a major cause of neonatal death and is a life-threatening risk factor for the mother; however, the underlying pathophysiology and prevention is still not clear 23. NLRP3 inflammasome activation induced by infection also causes preterm birth 24. Activation of NLRP3 inflammasome mediates infection- or inflammation-induced parturition 23, 24. In fact, many studies have shown that increased synthesis of IL-1β is associated with preterm delivery 25-29. In view of the evidence that overexpression of NLRP3 inflammasome and the release of its products may contribute to disease, the pharmacological inhibition of NLRP3 inflammation may trigger certain diseases.

The use of dietary plants and herbal preparations as alternative medicines has recently received considerable attention worldwide 30. *Cucurbita moschata* Duchesne (CMD), commonly known as “pumpkin”, is one such plant that has been cultivated throughout the world and various parts of CMD have been used traditionally as functional food or medicine 30. Studies reported that the fruit, fruit peel, stem, and seed extracts of CMD have anti-fatigue 31, wound-healing 32, anti-obesity 33, and anthelmintic 34 activities, respectively. Among them, dehydrodiconiferyl alcohol (DHCA), a lignan compound isolated from *C. moschata*, was suggested to exhibit anti-inflammatory effects by reducing NO production 35. The tendril of CMD (TCMD) water extract and the purified active compound rutin showed anti-inflammatory effects by inhibiting the production of pro-inflammatory cytokines such as IL-6 and TNF-α 36. However, the role of TCMD in regulating inflammasome activity is unknown. TCMD is one of the foods most pregnant women consumed traditionally in order to prevent spontaneous preterm birth because it alleviates swelling and prevents uterine contraction. However, there is no scientific basis for the effectiveness of TCMD in preventing preterm birth.

Regulation of IL-1β and inflammasome activity is important for effective treatment of inflammatory conditions including preterm birth. In this study, we thus investigated whether TCMD regulates NLRP3 inflammasome activation and IL-1β maturation in murine macrophages and human trophoblasts.

## Materials and Methods

### Animals and ethics

Wild type C57BL/6 male mice were purchased from Central Lab Animal Inc (Seoul, Korea). The animals were housed in the animal room at constant temperature (22‑24˚C) and maintained under a strict 12 h lighting cycle. Mice were sacrificed by cervical dislocation, and macrophages were obtained from femur and tibia. The animal study protocols were approved by the Institutional Animal Care and Use Committee of Chonnam National University (Approval No. CNU IACUC-YB-R-2019-61).

### Preparation of TCMD extract

Water extract of TCMD was prepared from dried tendrils of *C. moschata* Duch. TCMD (200 g) was added into 1 L of sterilized water and boiled at 90˚C for 4 h. This extract was filtered through filter papers (Whatman, Maidstone, UK), and concentrated using rotary evaporator (EYELA, Tokyo, Japan). The extract was lyophilized using a freeze dryer (Ilshin BioBase Co., Seoul, Korea) and stored at -20˚C. The powder was dissolved in phosphate buffered saline (PBS) and diluted to the appropriate concentrations with culture medium.

### Reagents

LPS, ATP, and monosodium urate crystals (MSU) were purchased from InvivoGen (San Diego, CA, USA). Nigericin sodium salt and rutin were purchased from Sigma-Aldrich (Sigma-Aldrich, St. Louis, MO, USA). Anti-β-actin antibody was obtained from Santa Cruz (Dallas, Texas, USA). Anti-IL-1β, anti-caspase-1, anti-pro-IL-1β, and anti-procaspase-1 were ordered from Cell Signaling Technology (Beverly, MA, USA).

### Preparation of murine macrophages and human trophoblast cells

BMDMs derived from murine bone marrow were prepared as previously described 37. Briefly, BMDMs were cultured in complete Iscove's modified Dulbecco's medium (IMDM, Gibco, Grand Island, NY, USA) including 30% L929 cell culture supernatant, 10% FBS, 1% sodium pyruvate, 1% MEM Non-Essential Amino Acids (MEM NEAA) and 1% penicillin/streptomycin in a 5% CO_2_ incubator at 37°C. After 3 days, 10 ml of the fresh medium was added and the cells were incubated for an additional 2 days. The cells were seeded in 48-well plates in triplicate at a concentration of 2×10^5^ cells/well for cytokine analysis or in 6-well plates at a concentration of 2×10^6^ cells/well for immunoblotting.

Sw.71 cells (a gift from Dr. Gil Mor, Yale University School of Medicine), representing a human trophoblast cell line, were cultured in DMEM (Welgene, Gyeongsangbuk-do, South Korea) containing 10% FBS (Corning, Manassas, VA, USA), 100 U/ml penicillin-streptomycin (Gibco, Grand Island, NY, USA), 1 mM HEPES, 0.1 mM non-essential amino acids and 1 mM sodium pyruvate. Cells were seeded in 48-well (5×10^4^ cells/well) plates for cytokine analysis and in 6-well (5×10^5^ cells/well) plates for real-time PCR analysis and incubated at 37°C and 5% CO_2_ overnight.

### Inflammasome activation or inhibition

BMDMs were layered on 48-well plates at a concentration of 1×10^6^ cells/ml for cytokine analysis or on 6-well plates at a concentration of 1×10^6^ cells/ml for immunoblotting analysis. BMDMs were pretreated with various doses of TCMD for 2 h after priming with LPS (100 ng/ml) for 6 h and subsequently treated with ATP (2 mM) or nigericin (10 μM) for 30 min or monosodium urate (MSU) (200 μg/ml) for 4 h. The culture supernatants were collected to measure the levels of IL-6 and IL-1β. Sw.71 cells were plated in 48-well plates at a concentration of 5×10^5^ cells/ml for cytokine analysis or 6-well plates at a concentration of 5×10^5^ cells/ml for Western blot.

### Measurement of cytokines

The concentrations of IL-6 and IL-1β in culture supernatants were measured using enzyme-linked immunosorbent assay (ELISA) kits (R&D Systems, Minneapolis, MN, USA) according to the manufacturer's instructions.

### Western blotting analysis

BMDMs and SW.71 cells were seeded into 6-well plates at a density of 1×10^6^ cells/ml and 5×10^5^ cells/ml, respectively and incubated overnight. BMDMs were primed with LPS (100 ng/ml) for 6 h prior to ATP (2 mM) or nigericin (20 μM) for 30 min or MSU (200 μg/ml) for 4 h. SW.71 cells were treated with MSU (100 μg/ml) for 72 h. Cells were lysed at the indicated time point in a buffer containing 1% Nonidet P-40, 50 mM Tris (pH 7.4), 250 mM NaCl, 5 mM EDTA, 50 mM NaF, 1 mM Na3VO4, and 0.02% NaN3 supplemented with protease inhibitor (complete, Mini, EDTA-free, Roche, Mannheim, Germany), phosphatase inhibitor (Phosphatase Inhibitor Cocktail 2, Sigma-Aldrich), and 2 mM dithiothreitol. In an experiment using BMDMs, cell lysates and culture supernatant were used in combination. However, in the experiment using SW.71 cells, cell lysates were used for detection of tested proteins except cleaved IL-1β. For the detection of cleaved IL-1β, culture supernatant was used. Protein samples were separated by 10%, 12%, and 15% sodium dodecyl sulfate polyacrylamide gel electrophoresis (SDS-PAGE) and transferred onto nitrocellulose membranes. The membranes were blocked with 5% skim milk for 1 h at room temperature and probed with primary antibodies against pro-IL-1β, IL-1β, pro-caspase-1, caspase-1, and β-actin overnight at 4 °C. The membranes were incubated with relevant secondary antibodies (Santa Cruz biotechnology) for 2 h at room temperature and proteins were detected using the ECL substrate (Bio-Rad, Hercules, CA, USA).

### Lactate dehydrogenase assay

The release of lactate dehydrogenase (LDH) into the culture medium was determined using the LDH Cytotoxicity Assay Kit (Promega, Madison, WI) according to the manufacturer's instructions.

### ASC oligomerization assay

After inflammasome activation in the absence or presence of TCMD extracts, the cells were harvested, resuspended in cold lysis buffer containing Triton X-100 and complete protease inhibitor cocktail (Roche, Mannheim, Germany), and passed 10 times through a 27-gauge syringe. After centrifugation at 5000 rpm for 10 min, the supernatants (Triton-soluble fraction) were mixed with sample loading buffer (5×) and used to detect ASC (Cell Signaling Technology, Cat No. 67824) and β-actin (Santa Cruz Biotechnology, sc-47778). The remaining cell pellets were resuspended in PBS buffer containing 2 mM disuccinimidyl suberate (DSS, Sigma-Aldrich) cross-linker and were incubated at room temperature for 30 min, followed by centrifugation at 5000 rpm for 10 min. To detect ASC oligomerization, the cross-linked pellets (Triton-insoluble fraction) were separated on 12% SDS-PAGE and transferred to NC membranes. These membranes were probed with primary antibodies against ASC. After immunoblotting with HRP-conjugated goat anti-rabbit IgG (H+L) (Invitrogen) or goat anti-mouse IgG (H+L) secondary antibodies (Invitrogen), the proteins were detected using Clarity Western ECL Substrate (Bio-Rad).

Cells were harvested after inflammasome activation with or without TCMD extract, resuspended in 0.5 ml PBS buffer, and passed 10 times through a 27-gauge syringe. Cell lysates were centrifuged at 1000 rpm for 10 min to collect the cells.

Supernatants were diluted with equal volumes of lysis buffer containing triton X-100 and complete protease inhibitor cocktail (Roche, Mannheim, Germany), and centrifuged at 5000 rpm for another 10 min to pellet the ASC pyroptosomes. The pellets were resuspended in PBS buffer containing 2 mM disuccinimidyl suberate (DSS) cross-linker and were incubated at room temperature for 30 min followed by centrifugation at 5000 rpm for 10 min. The cross-linked pellets were fractionated on 12% SDS-PAGE, and ASC oligomerization was assessed by immunoblotting with ASC antibody (Cell signaling Technology; Cat No. 67824).

### Statistical analysis

The statistical significance of differences between groups was determined via a two-tailed Student's t-test or one-way analysis of variance (ANOVA) followed by Bonferroni post hoc analysis (GraphPad Prism 5; GraphPad Software Inc., La Jolla, CA, USA). P-Values < 0.05 were considered significant.

## Results

### TCMD inhibits inflammasome-induced IL-1β secretion in macrophages

**Figure 1:**
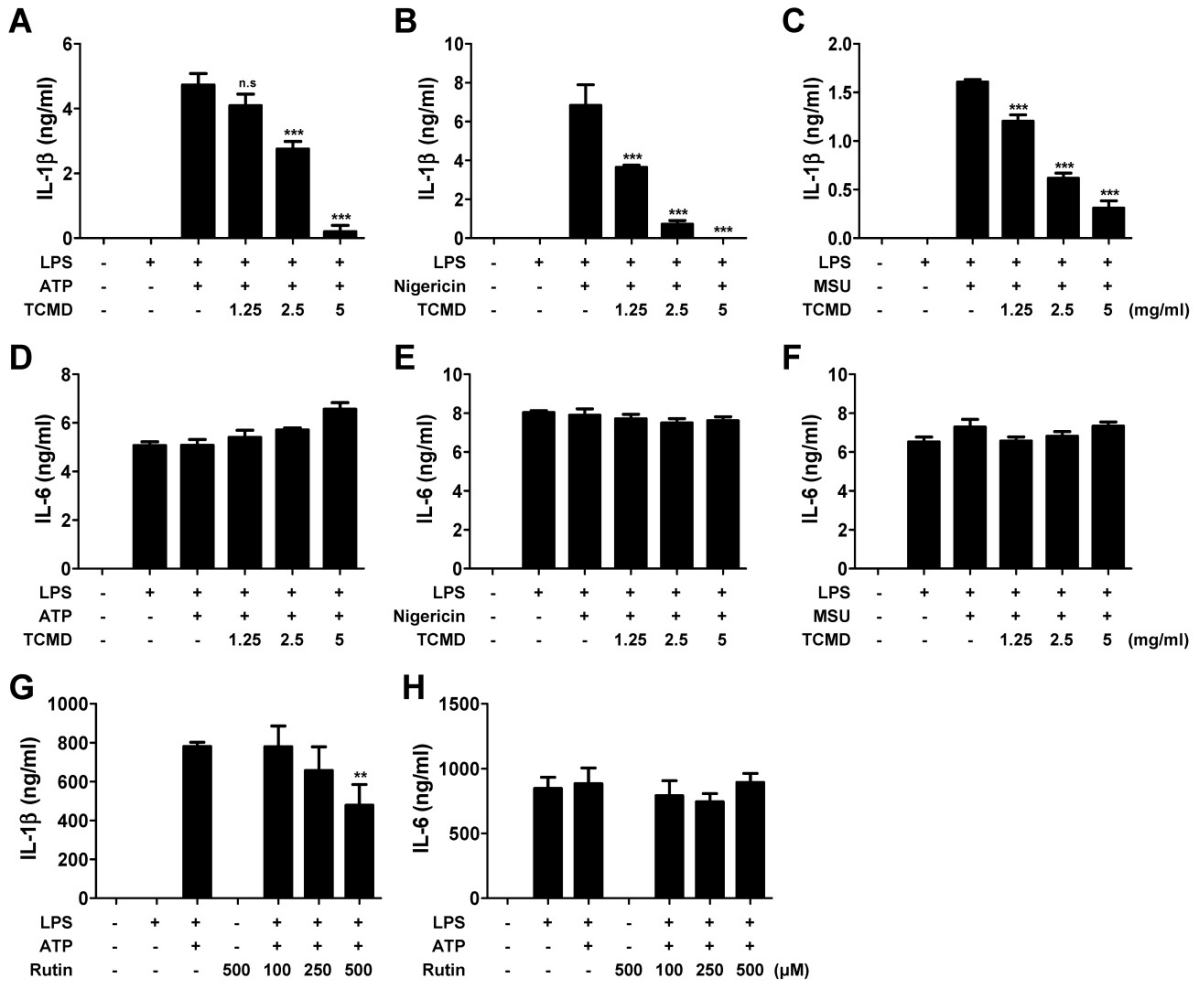
** TCMD inhibits IL-1β secretion induced by NLRP3 inflammasome activation in BMDMs.** BMDMs were primed with LPS (100 ng/ml) for 6 h prior to TCMD (1.25, 2.5, and 5 mg/ml) (A-F) or rutin (100, 250, and 500 μM) (G and H) treatment for 2 h, and subsequently treated with 2 mM ATP for 30 min (A and D), or 10 μM nigericin for 30 min (B and E), or 200 μg/ml MSU for 4 h (C and F). The levels of IL-1β (A-C and G) and IL-6 (D-F and H) in culture supernatants were measured by ELISA. The results are from one experiment that is representative of three independent experiments and expressed as means ± SD. ***P < 0.001.

We first investigated whether TCMD inhibited proinflammatory cytokines such as IL-6 and IL-1β. The IL-1β secretion was significantly increased in LPS-primed BMDMs in response to ATP, nigericin, and MSU and these increases were inhibited by TCMD in a dose-dependent manner (Figure 1A-1C). We investigated whether TCMD specifically inhibited IL-1β release by analyzing the release of another pro-inflammatory cytokine, IL-6, in the same culture supernatants. However, pre-treatment of cells with TCMD had no inhibitory effect on IL-6 release (Figure 1D-1F). A previous study revealed that rutin is a purified active compound in water extract of TCMD 36. Accordingly, we further investigated whether rutin influences IL-1β secretion. As shown in Figure 1G and 1H, rutin slightly decreased ATP-induced secretion of IL-1β, but not IL-6, in LPS-primed BMDMs in a dose-dependent manner. These findings show that TCMD suppresses the secretion of IL-1β but not IL-6 in activated macrophages, indicating that TCMD specifically inhibits inflammasome response.

### TCMD inhibits pyroptosis by inhibiting LDH release in macrophages

**Figure 2:**
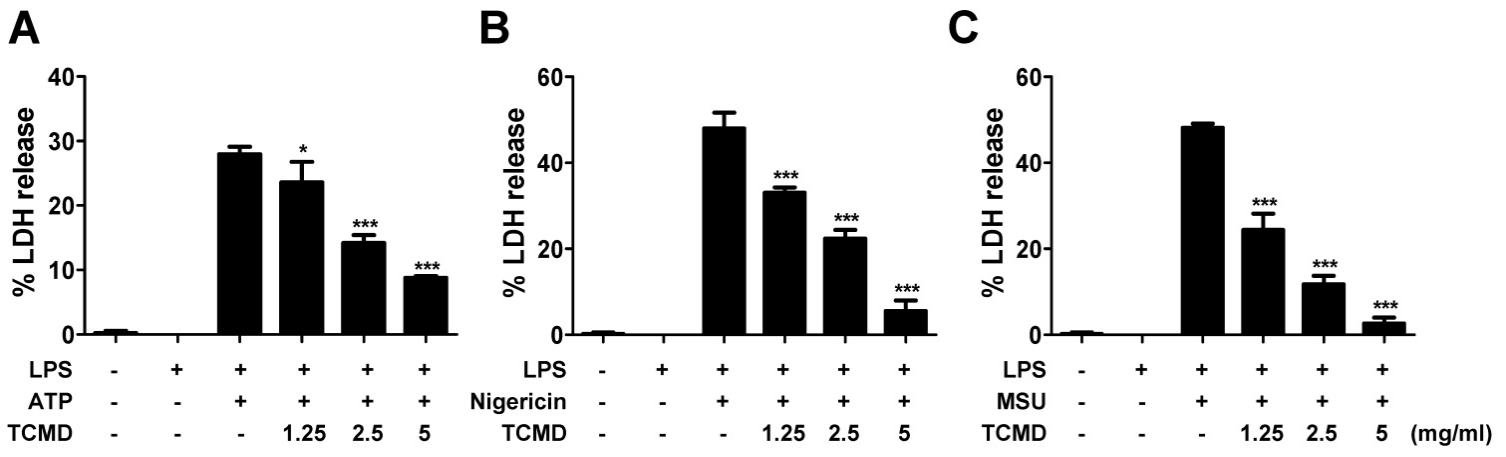
** TCMD inhibits LDH release induced by NLRP3 inflammasome activation in BMDMs.** BMDMs were primed with LPS (100 ng/ml) for 6 h prior to TCMD (1.25, 2.5, and 5 mg/ml) treatment for 2 h (A-C), and subsequently treated with 2 mM ATP for 30 min (A), or 10 μM nigericin for 30 min (B), or 200 μg/ml MSU for 4 h (C). The culture supernatants were collected for LDH determination. The results are from one experiment that is representative of three independent experiments and expressed as means ± SD. ***P < 0.001.

NLRP3 inflammasome activation also induces inflammatory cell death known as pyroptosis 38. Pyroptotic cell death was assessed by measuring the LDH release after activating NLRP3 inflammasome in BMDMs and the effect of TCMD was evaluated. TCMD prevented ATP-induced LDH release in a dose-dependent manner in LPS-primed BMDMs (Figure 2A). Moreover, LDH release induced by nigericin and MSU was also inhibited by TCMD (Figure 2B, 2C). These results suggest that TCMD can suppress pyroptosis via inhibition of NLRP3 inflammasome activation.

### TCMD inhibits cleavage of IL-1β and caspase-1 in macrophages in response to ATP, nigericin, and MSU

**Figure 3:**
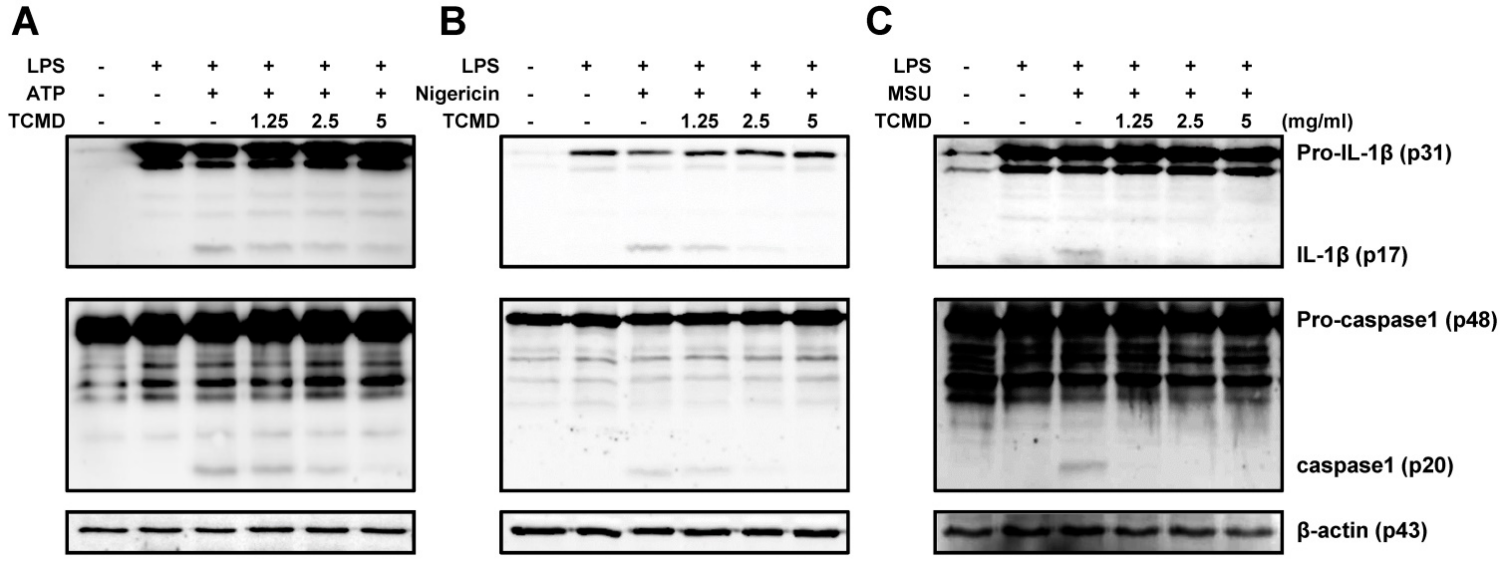
** TCMD inhibits production of IL-1β and activation of caspase-1 induced by NLRP3 inflammasome activation in BMDMs.** BMDMs were primed with LPS (100 ng/ml) for 6 h prior to TCMD (1.25, 2.5, and 5 mg/ml) treatment for 2 h (A-C), and subsequently treated with 2 mM ATP for 30 min (A), or 10 μM nigericin for 30 min (B), or 200 μg/ml MSU for 4 h (C). Pro- and cleaved-forms of IL-1β and caspase-1 were detected by Western blot analysis. β-actin was used as a control for the loading volume. The results are from one experiment that is representative of two independent experiments.

Maturation of pro-IL-1β to induce inflammation requires caspase-1 activation. Therefore, we evaluated whether TCMD regulates caspase-1 activation and IL-1β maturation using Western blot analysis. The cleavage of IL-1β and caspase-1 induced by ATP, nigericin, and MSU was significantly reduced by TCMD in the culture supernatants (Figure 3A-3C). Unlike the cleavage forms in the supernatants, the expression of pro-IL-1β and pro-caspase-1 in the cell lysates was not changed by TCMD treatment (Figure 3A-3C). These results suggest that TCMD may regulates IL-1β secretion by inhibiting caspase-1 activation.

### TCMD inhibits the oligomerization of ASC in macrophages

**Figure 4:**
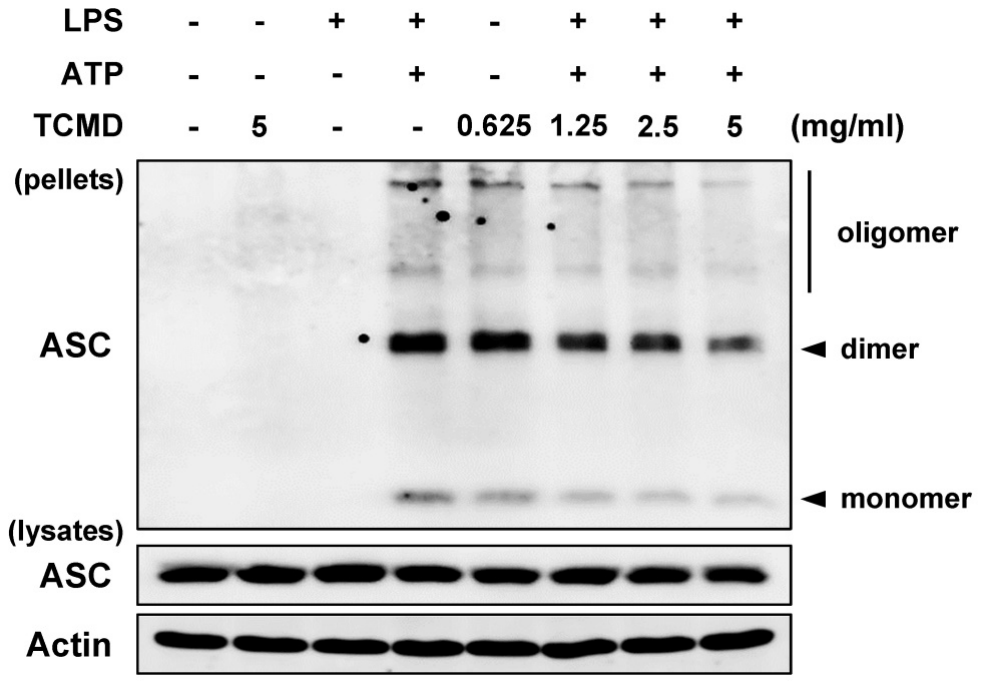
** TCMD inhibits ASC oligomerization induced by NLRP3 inflammasome activation in BMDMs.** BMDMs were primed with LPS (100 ng/ml) for 6 h prior to TCMD (0.625, 1.25, 2.5, and 5 mg/ml) treatment for 2 h, and subsequently treated with 2 mM ATP for 30 min. ASC oligomerization was detected by Western blot analysis. β-actin was used as a control for the loading volume. The results are from one experiment that is representative of two independent experiments.

A previous study reported that IL-1β secretion is induced by activated caspase-1 via assembly of an inflammasome complex composed of NLRP3, oligomerized ASC, and caspase-1 39. Moreover, ASCs form a dimer or oligomer in inflammasome-activated conditions 40. We thus examined the impact of TCMD on ASC oligomerization in BMDMs, which are regarded as the hallmarks of inflammasome formation. Western blot analysis revealed that ASC oligomerization induced by ATP was decreased by TCMD treatment in a dose-dependent manner (Figure 4), suggesting that TCMD has the potential to inhibit inflammasome activation via regulation of ASC oligomerization.

### TCMD inhibits IL-1β secretion and LDH release in human trophoblasts

**Figure 5:**
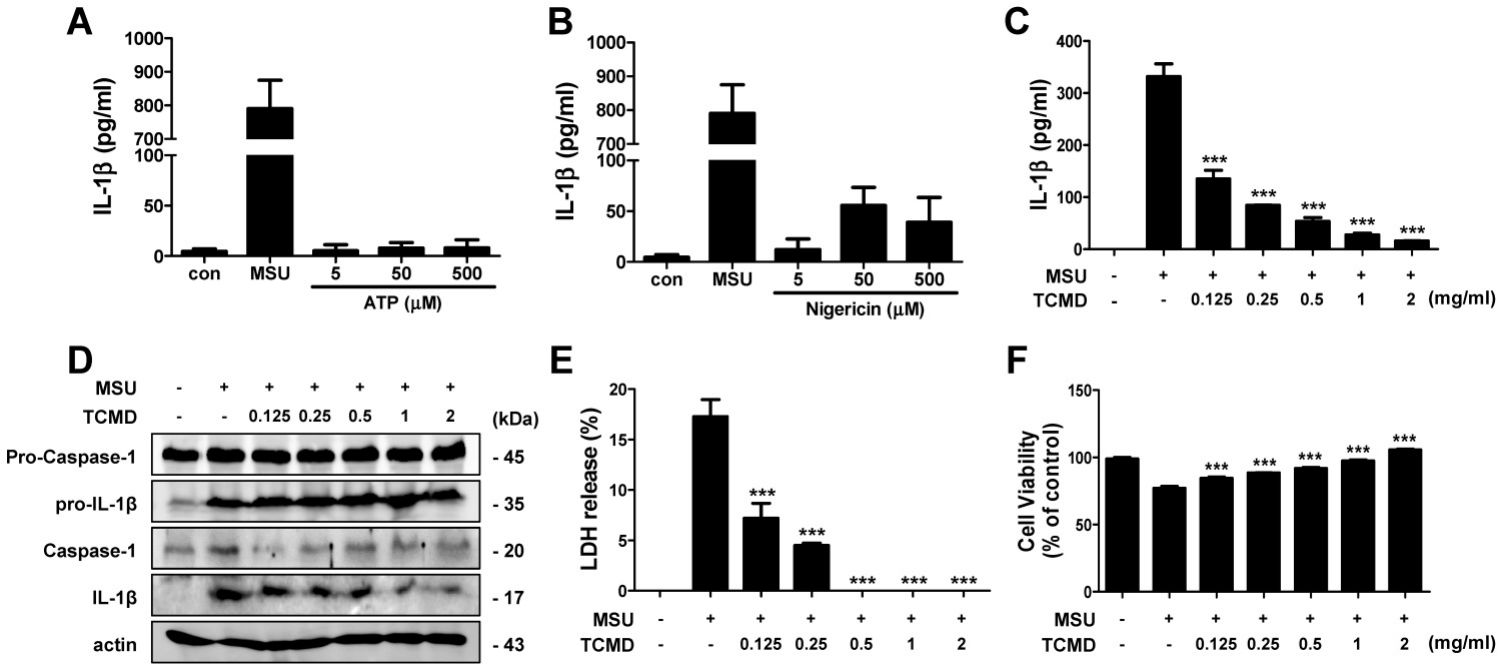
** TCMD inhibits IL-1β secretion and LDH release induced by NLRP3 inflammasome activation in human trophoblasts.** SW.71 cells were treated with ATP (5-500 μM), nigericin (0.05-5 μM), and MSU (100 μg/ml) for 72 h (A and B). The cells were pretreated with TCMD (0.125, 0.25, 0.5, 1, and 2 mg/ml) for 2 h and activated with MSU (100 μg/ml) for 72 h (A-F). The levels of IL-1β in culture supernatants were measured by ELISA (A-C). Pro- and cleaved-forms of IL-1β were detected by western blot analysis (D). LDH release was measured in culture supernatants (E). Cell viability was measured by MTT assay (F). β-actin was used as a control for the loading volume. The results are from one experiment that is representative of two (D) or three (A-C, E, F) independent experiments and expressed as means ± SD. *P < 0.05, **P < 0.01, ***P < 0.001.

In a dose-dependent experiment, we found that ATP treatment did not induce IL-1β secretion in SW.71 cells at ranges of 5-500 μM and nigericin could induce only small amount secretion of IL-1β at doses of 0.5 and 5 μM (Figure 5A, 5B). In contrast, MSU (100 μg/ml) could produce huge amount of IL-1β in the cells, even without LPS priming (Figure 5A, 5B), which is consistent with a previous study 41. To determine whether TCMD inhibits IL-1β secretion in human trophoblasts, we administered TCMD prior to MSU activation and measured the release of IL-1β by ELISA and Western blot. The MSU-stimulated IL-1β secretion was significantly down-regulated by TCMD pre-treatment in a dose-dependent manner (Figure 5C). In addition, cleaved caspase-1 and IL-1β was also reduced by TCMD in SW.71 cells in response to MSU (Figure 5D). TCMD treatment also decreased LDH release and improved cell viability (Figure 5E, 5F). Taken together, TCMD inhibits NLRP3 inflammasome activity and pyroptotic cell death in human trophoblasts.

## Discussion

Inflammasome activity is important for the maturation and secretion of IL-1β, which is involved in the pathophysiology of tissue damage 42-46. Increased levels of IL-1β have a detrimental effect on pregnancy, leading to complications such as chorioamnionitis, preeclampsia, and preterm birth 47-50. CMD has been widely used in traditional herbal remedies and foods and has received considerable attention due to its extensive bioactivity as an anti-diabetic 51, anti-cancer 52, anti-obesity 53, and anti-inflammatory agent 35. However, the efficacy of TCMD has not been fully understood. In the present study, we found that TCMD plays a crucial role in inhibiting NLRP3 inflammasome activation in murine macrophages and human trophoblast cells. We suggest that TCMD is a potential food and therapeutic agent in the prevention of inflammatory conditions including preterm labor.

Our results showed that TCMD pretreatment significantly inhibited IL-1β synthesis by ATP, nigericin, and MSU in LPS-primed BMDMs. However, TCMD had no effect on the release of IL-6, which indicates that TCMD specifically regulates NLRP3 inflammasome activation. In addition to the secretion and maturation of IL-1β, the inflammasome activation triggers programmed, pro-inflammatory cell death known as pyroptosis 20. Pyroptosis is mediated via caspase-1 activation inducing the formation of membrane pores, cellular swelling followed by membrane destruction, and release of intracellular content into the extracellular space, including cytoplasmic proteins such as LDH 54. Our results indicated that TCMD inhibits LDH release, suggesting that TCMD can potentially prevent pyroptotic cell death in macrophages.

Many ASC molecular events related to inflammasome activation have been reported 55. The adapter protein ASC is essential for both NLRP3 and AIM2 inflammasome formation 56, 57. Therefore, investigation of ASC oligomerization is regarded as a hallmark of inflammasome formation 55. In a previous study, *Artemisia princeps* extract (APO) inhibited ASC oligomerization triggered by ATP-, silica-, or nigericin-induced NLRP3 inflammasomes and poly (dA:dT)-induced AIM2 inflammasome activation 55. Consistent with that study, our results indicate that the ASC oligomerization was increased in the LPS-primed BMDMs activated by ATP and was dose-dependently inhibited by TCMD (Figure 4). These results indicate that TCMD may suppress inflammatory activation via regulation of ASC oligomerization.

In the presence of hypertension, the risk of preterm birth was increased with increasing uric acid levels 58. Women with hypertension and hyperuricemia had a 3.6-week shorter gestation period and a 7-fold higher prevalence of premature birth than women with hypertension alone 58. Therefore, an increase in uric acid level during pregnancy may induce preterm birth. Preterm birth is also associated with placental infection and trophoblast inflammation 24. In trophoblasts, NLRP3 inflammasome activation and elevated levels of proinflammatory cytokines including IL-1β have been associated with pregnancy complications, such as preeclampsia, miscarriage, and preterm birth 23, 26. Therefore, the regulation of intra-placental inflammation via extracellular or intracellular stimulation may prevent preterm labor and stillbirth. Because first trimester trophoblast cells express pattern recognition receptors (PRRs) such as TLRs and Nod proteins, stimulation by bacteria and viruses induces inflammatory response 41. A previous study demonstrated that treatment with antiphospholipid antibodies (aPL) via TLR4 activation raises the uric acid levels in human trophoblasts, which in turn activate the Nalp3/ASC inflammasome inducing IL-1β secretion 26. This mechanism may trigger placental dysfunction and exacerbate the pregnancy outcome of patients with antiphospholipid syndrome (APS) 26. Untreated first trimester trophoblast cells express pro-IL-1β at a high level and therefore MSU acting as DAMP to activate NLRP3 inflammasome, was treated with SW.71 cells 23, 26, 41. Several studies have demonstrated that MSU induces IL-1β secretion by activating inflammasome in trophoblasts 24, 41. Our results consistently showed that MSU treatment induces IL-1β secretion in SW.71 cells, which was significantly inhibited by TCMD. TCMD also inhibits MSU-induced LDH release and restores cell viability in SW.71 cells.

Overall, TCMD exerts anti-inflammatory effects by inhibiting IL-1β secretion in BMDMs and human trophoblast cells. However, TCMD had no effect on the expression of pro-IL-1β, and procaspase-1, and ASC, which indicates that TCMD specifically inhibits inflammasome activation. These results indicate that TCMD acts as a direct inhibitor of the NLRP3 inflammasome. This report is the first of its kind to provide scientific evidence suggesting that TCMD is a highly effective natural extract that can be used to modulate the inflammatory response via inhibition of NLRP3 inflammasome activation.

In conclusion, our present data indicate that TCMD is a potential anti-inflammatory agent that can be developed further for the treatment of inflammatory disorders and preterm birth. Therefore, additional in vivo studies are needed to further confirm the efficacy of TCMD in pregnant women and fetuses.

## Abbreviations

CMD: *Cucurbita moschata* Duchesne
TCMD: tendrils of *Cucurbita moschata* Duchesne
BMDMs: bone marrow-derived macrophages
MSU: monosodium urate
IBD: inflammatory bowel disease
DHCA: dehydrodiconiferyl alcohol
PBS: phosphate buffered saline
MEM NEAA: MEM Non-Essential Amino Acids
ELISA: enzyme-linked immunosorbent assay
LDH: lactate dehydrogenase
DSS: disuccinimidyl suberate
APO: *Artemisia princeps* extract
PRRs: pattern recognition receptors
aPL: antiphospholipid antibodies
APS: antiphospholipid syndrome
